# Effect of phase-change material blood containers on the quality of red blood cells during transportation in environmentally-challenging conditions

**DOI:** 10.1371/journal.pone.0227862

**Published:** 2020-01-29

**Authors:** Xiaoyang Yi, Minxia Liu, Jiexi Wang, Qun Luo, Hailong Zhuo, Shaoduo Yan, Donggen Wang, Ying Han

**Affiliations:** 1 Beijing Key Laboratory of Blood Safety Supply Technologies, Key Laboratory of Blood Safety Supply Technologies of PLA, Institute of Transfusion Medicine, Academy of Military Medical Science, Beijing, China; 2 Transfusion Department, Beijing, China; Massachusetts Institute of Technology, UNITED STATES

## Abstract

**Background:**

The effect of phase-change material blood containers on the quality of stored red blood cells (RBCs) transported in the Qinghai-Tibet Plateau remains to be studied.

**Study design and methods:**

RBCs stored in a phase-change material blood container were transported from Chengdu to Tibet and then back to Chengdu. The detection time points were the 1^st^ day of fresh-collected RBCs (group 1), the 14^th^ day of resting refrigerated storage (group 2), and the 14^th^ day of plateau transportation under refrigerated storage in the container (group 3). RBC counts, hemoglobin (HGB) content, free hemoglobin (FHb) content, blood biochemical indexes, hemorheologic indexes and 2,3-DPG content were detected.

**Results:**

Compared with group 2, RBC counts and HGB were decreased, and the mean corpuscular volume (MCV), FHb and K^+^ content were increased in group 3. The glucose consumption and lactic acid production were significantly increased in groups 2 and 3. Compared with group 2, the 2,3-DPG content and whole blood viscosity were decreased in group 3. After resting refrigerated storage and plateau transportation, the RBC quality still met the national standard (GB18469-2012 whole blood and component blood quality requirements).

**Conclusion:**

The phase-change material blood container can be maintained at a constant temperature under plateau environmental conditions, ensuring that the quality of the stored RBCs is compliant with GB18469-2012 whole blood and component blood quality requirements.

## Introduction

The environmental characteristics of Qinghai-Tibet Plateau are low pressure and low oxygen. For example, the altitude of Lhasa in Tibet is 3650 m, the atmospheric pressure is approximately 66% of that in the plain, and the oxygen content is approximately 70% of that in the plain[1]. Due to the long border, complex terrain (mainly occluded with mountains and hills) and poor road conditions, traffic disruption often occurs. Thus, three-dimensional traffic was initially developed in Tibet. Transportation was mainly based on highways. The medical supplies were affected by objective factors, such as transportation difficulties and immediate acquisition.

As a result, it was difficult to ensure timely blood supply. Blood products undergo hemolysis easily when transported for long periods of time because of the bumpy road. Especially when met with externally bad weather, such as temperatures above 30°C or below -20°C, blood products are extremely vulnerable. Therefore, it is very important to maintain the refrigerated temperature of blood during transportation[2]. In addition, it is difficult to ensure a stable power supply during transportation, and power supply equipment, such as batteries, is too cumbersome, and logistical and transport difficulties are increased. Therefore, it is necessary to develop blood transport equipment that does not require electricity supply and has a long holding time to provide logistical and possibly clinical benefits.

**Fig 1:**
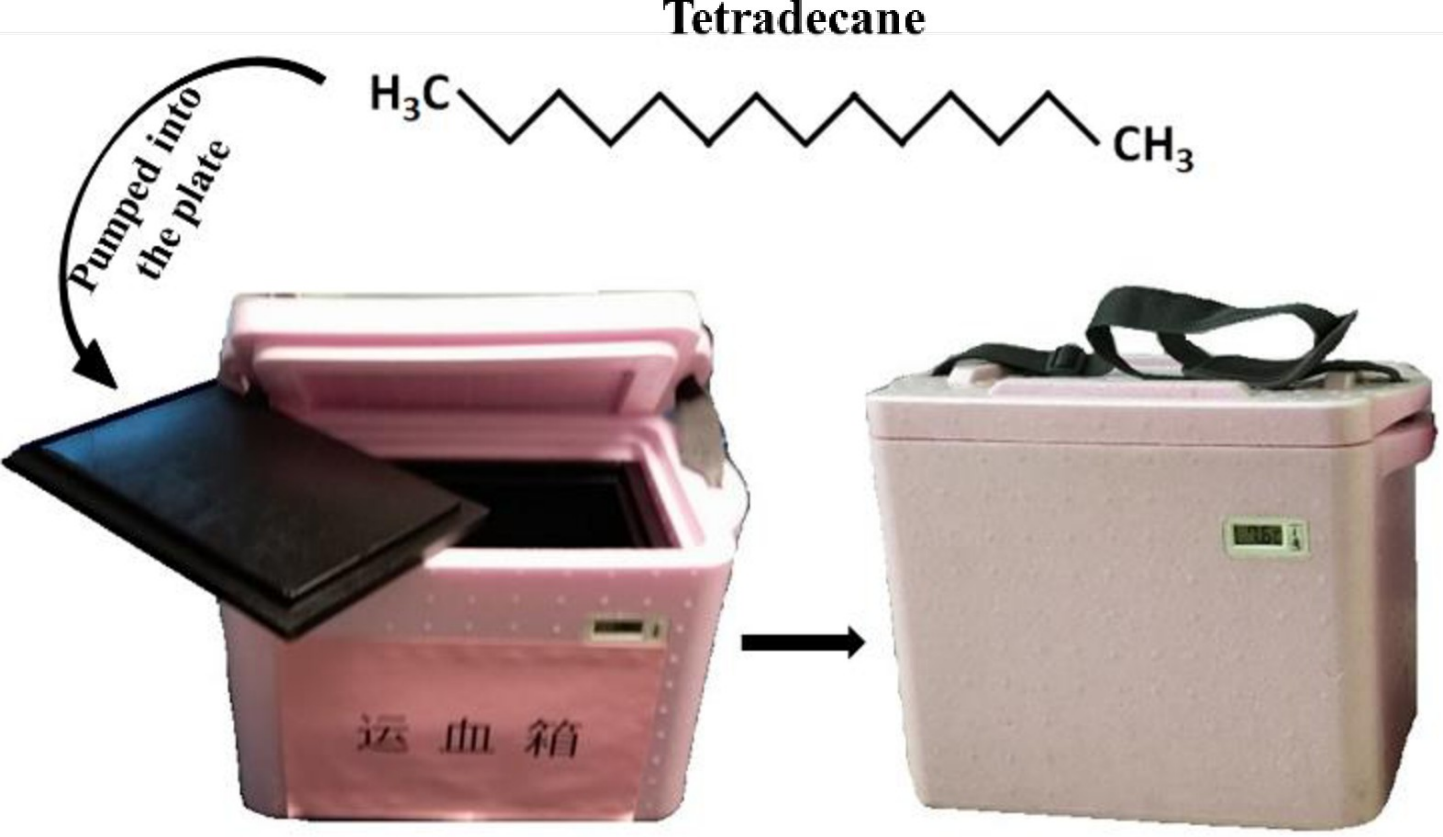
The structure and appearance of phase-change material blood containers.

Our research team designed and developed a phase-change material transport container without a power supply. The thermal insulation material mainly consists of tetradecane, and its phase transition temperature is 4.5~5.6°C. Heat and exothermic cycles are used to adjust the heat balance absorbed through the phase change material into the container at a specific temperature, which ensures that the inside of the chamber can be maintained at a suitable temperature (1 to 10°C) for a longer period of time (≥60 h) when the ambient temperature changes. The structure and appearance of phase-change material blood containers are shown in Fig 1. AABB standards and FDA guidelines both state that RBCs should only be transfused if they have been stored between 1 and 6°C and shipped between 1 and 10°C, and RBCs exposed to temperatures above 10°C are not necessarily unsuitable for transfusion[3–5]. A phase-change material transport container can ensure that the RBCs are safely transported at the FDA-specified temperature[6,7]. There are no related guidelines in China at present.

Whether the present RBC preservation method is suitable for plateaus in a high-altitude population and the effect of phase-change material blood containers on the quality of stored RBCs in the plateau remains to be studied. Therefore, the aim of this study was mainly to evaluate the quality of RBCs after plateau transportation by assessing RBC counts, hemoglobin (HGB) content, free hemoglobin (FHb) content, blood biochemical index, hemorheologic indexes and 2,3-DPG content and to clarify the role of phase-change material blood containers in blood transport in the plateau.

## Materials and methods

### Preparation and transportation of RBCs

The study was approved by the Ethics Committee of the Academy of Military Medical Sciences and all aspects of the study comply with the Declaration of Helsinki. Ethics Committee of the Academy of Military Medical Sciences specifically approved that no informed consent was required because data were going to be analyzed anonymously. All donations were obtained from eligible, voluntary donors without minors.

Suspended RBCs (400ml/bag, n = 5) and leukocyte-depleted suspended RBCs (300ml/bag, n = 5) were obtained from the Chengdu Military General Hospital. The units were subjected to the following treatment conditions: resting refrigerated storage (150~200ml/bag) and transportation under refrigerated storage (150~200ml/bag). The detection time points were the 1^st^ day of fresh-collected RBCs (group 1), the 14^th^ day of resting refrigerated storage (group 2), and the 14^th^ day of plateau transportation under refrigerated storage in the container (group 3). The transportation route for group 3 was Chengdu (1^st^ day~5^th^ day)→ Lhasa (6^th^ day ~8^th^ day)→ Shannan (9^th^ day ~ 10^th^ day)→ Chengdu (11^th^ day ~14^th^ day). The blood samples were unpacked from the shipping containers to refrigerator and repacked into the shipping container at the transit points, and were firmly packed to prevent excessive disturbance within the containers. RBC concentrates were prepared in PVC bags (Nagele, Sichuan, China) from whole blood collected in CPDA (Nagele). WBC filters were obtained from Nagele.

### In vitro quality analysis

RBC counts, HGB, mean corpuscular volume (MCV) and hematocrit (HCT) were determined by a hematology analyzer (Pentra 80, France Horiba ABX). FHb was measured using a test kit according to the manufacturer’s instructions (Beijing Ruierda Biotechnology). The blood biochemical indexes were measured using an automatic blood biochemical analyzer (GEM premier 3000, USA Instrumentation Laboratory). The 2, 3-DPG content was measured using a kit according to the manufacturer’s instructions (Nanjing Senbeijia Biological Technology). RBC rheology detection was determined by an automated blood rheology analyzer (SA-9000, Beijing Succeeder). The inner temperature of the blood container was measured by temperature logger (RC-4, Zhejiang Elitech). The logger and its probe were inserted into the container.

### Statistical analysis

The results are expressed as the means ± standard deviation (SD). The effect of group 1 *versus* group 2/3 and group 2 *versus* group 3 were assessed using a two-way repeated-measures ANOVA. A p value of less than 0.05 was considered to be significant.

## Results

### Altitude, ambient temperature and inner temperature of the blood container during transportation

**Fig 2:**
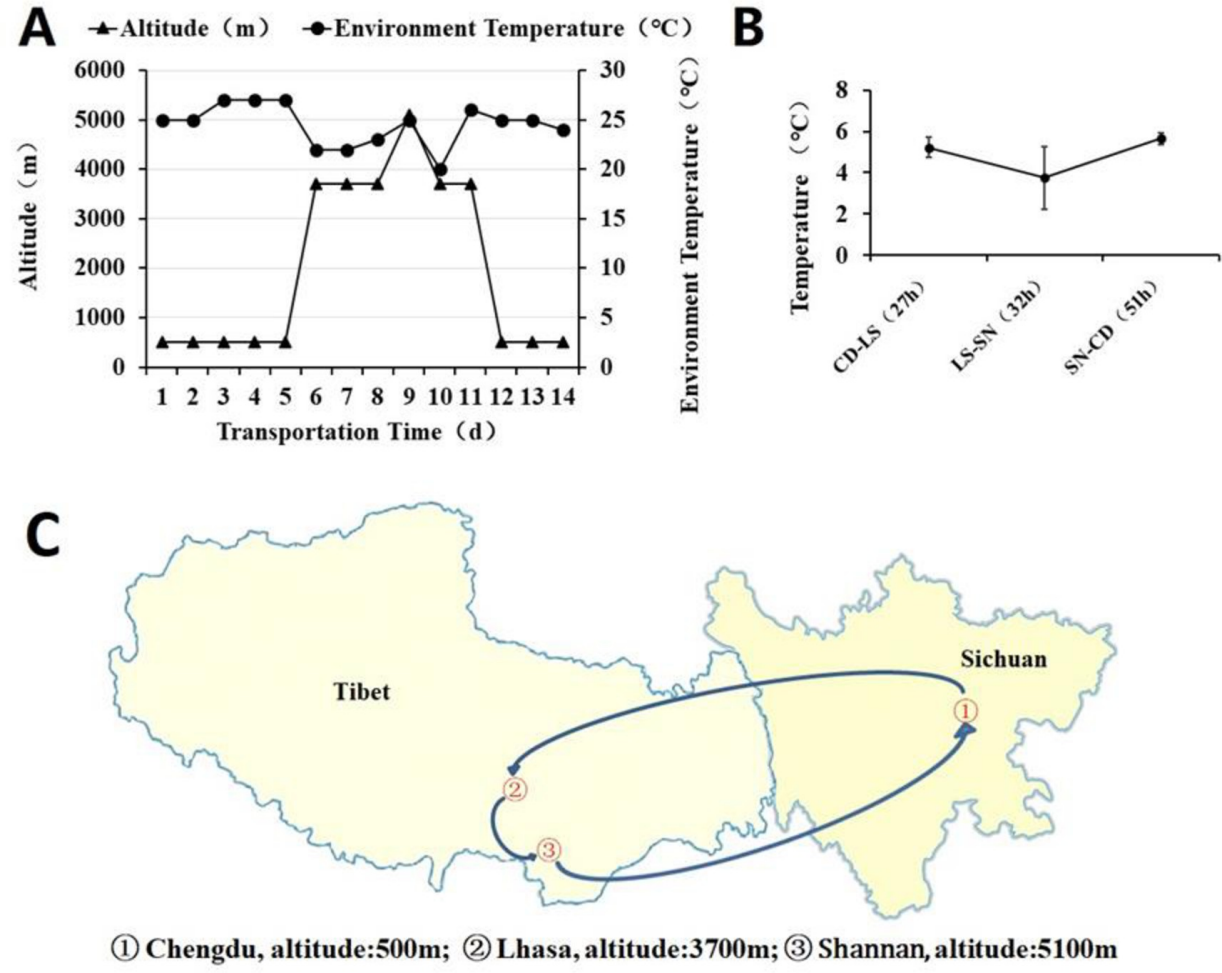
Altitude and ambient temperature where blood samples pass by were recorded every day (A). The transport time in the blood containers was approximately 27 h from Chengdu to Lhasa by air (CD-LS), 32 h from Lhasa to Shannan by road (LS-SN) and 51 h from Shannan to Chengdu by air (SN-CD). Blood samples were kept in the refrigerator at the rest of the time. The inner temperature of the blood container was recorded by a temperature logger every 15 minutes (B). A topographical map was shown (C).

First, blood samples were transported from Chengdu at an altitude of 500 m and then to Lhasa at an altitude of 3700 m. The highest altitude during transportation from Lhasa to Shannan was 5077 m, and the lowest altitude was 3021 m. Finally, the samples were transported from Shannan back to Chengdu. The ambient temperature ranged from 20°C to 27°C (Fig 2A). The transport time in the blood containers was approximately 27 h from Chengdu to Lhasa by air (CD-LS), 32 h from Lhasa to Shannan by road (LS-SN) and 51 h from Shannan to Chengdu by air (SN-CD) (Fig 2C). The transport time included time at the airport waiting for the flight. The inner temperature of the blood container was recorded by a temperature logger every 15 minutes. The inner temperatures were 5.23±0.47°C (CD-LS), (3.76 ± 1.51°C (LS-SN) and (5.65 ± 0.27°C (SN-CD), in accordance with FDA requirements specifying that the RBC transport temperature should range from 1°C to 10°C (Fig 2B).

### RBC counts, Hb, MCV, and HCT

**Fig 3:**
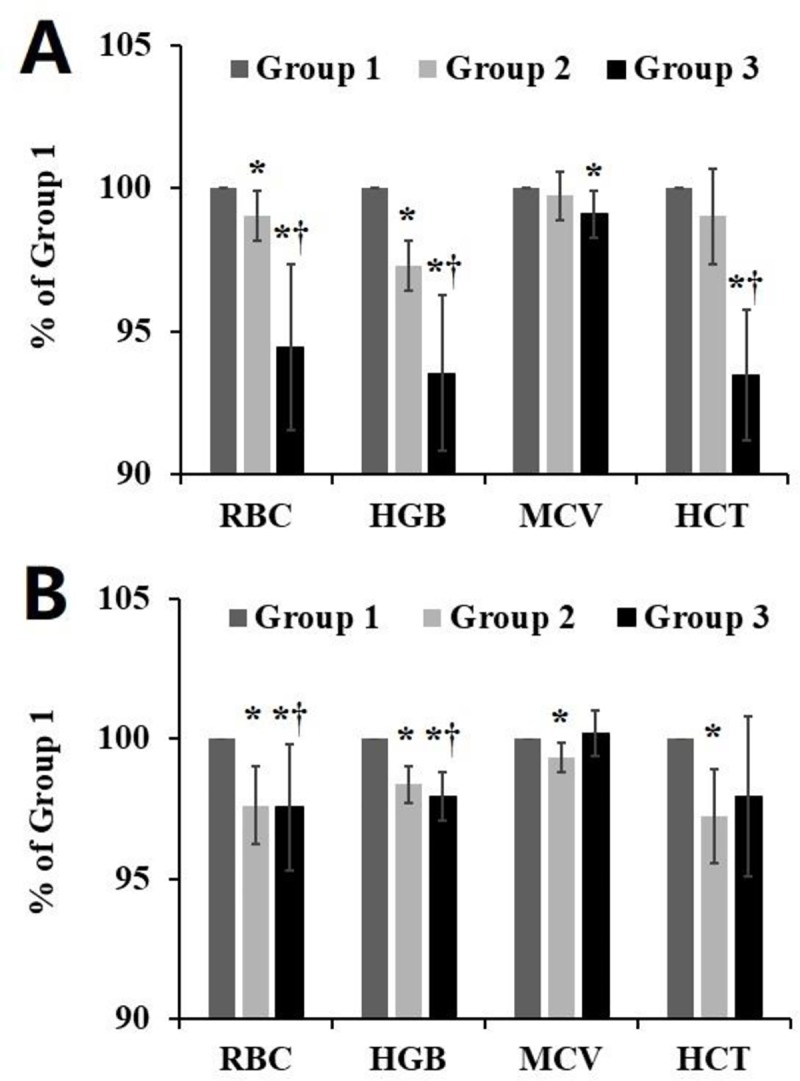
RBC counts, HGB, MCV and HCT of suspended RBCs (A) and leukocyte-depleted suspended RBCs (B) were determined by a hematology analyzer. Group 1 were the 1^st^ day fresh-collected RBCs, group 2 was the 14^th^ day RBCs with resting refrigerated storage, and group 3 was the 14^th^ day RBCs after plateau transportation. The data represent mean ± SD from five RBCs samples in each group. *p < 0.05 compared to group 1; †p < 0.05 compared to group 2.

Among the suspended RBCs (Fig 3A), the RBC count in group 1 was (6.90±0.70)×10^12^/L, and after storage for 14 days in Chengdu the RBC count was (6.83±0.70)×10^12^/L. The count decreased to (6.53 ± 0.81) × 10^12^ /L after plateau transportation. The Hb content in group 1, group 2 and group 3 was 190.4 ± 4.67 g/L, 185.2 ± 4.17 g/L and 178 ± 2.83 g/L, respectively. Over the 14-day storage period, the RBC concentration in group 2 was decreased by approximately 1% and that in group 3 was decreased by approximately 5.4%. There was a significant difference between group 2 and group 3 (Fig 3A). There was no significant difference in the MCV between group 1, group 2 and group 3. The HCT in group 3 was significantly lower than that in group 1 and group 2 (p<0.05).

Among the leukocyte-depleted RBCs (Fig 3B), the RBC counts in group 1, group 2 and group 3 were (6.23±0.24)×10^12^/L, (6.13±0.23)×10^12^/L and (6.12 ± 0.6) × 10^12^ /L, respectively. The Hb content in group 1, group 2 and group 3 was 184 ± 9.21 g/L, 181 ± 9.06 g/L and 180.2 ± 8.84 g/L, respectively. There was no significant difference in the RBC concentration and Hb content between group 2 and group 3. The MCV in group 2 was significantly lower than that in group 1 and group 3 (p<0.05). The HCT in group 2 and group 3 was significantly lower than that in group 1, but there was no significant difference between group 2 and group 3.

### Analysis of the FHb content

**Fig 4:**
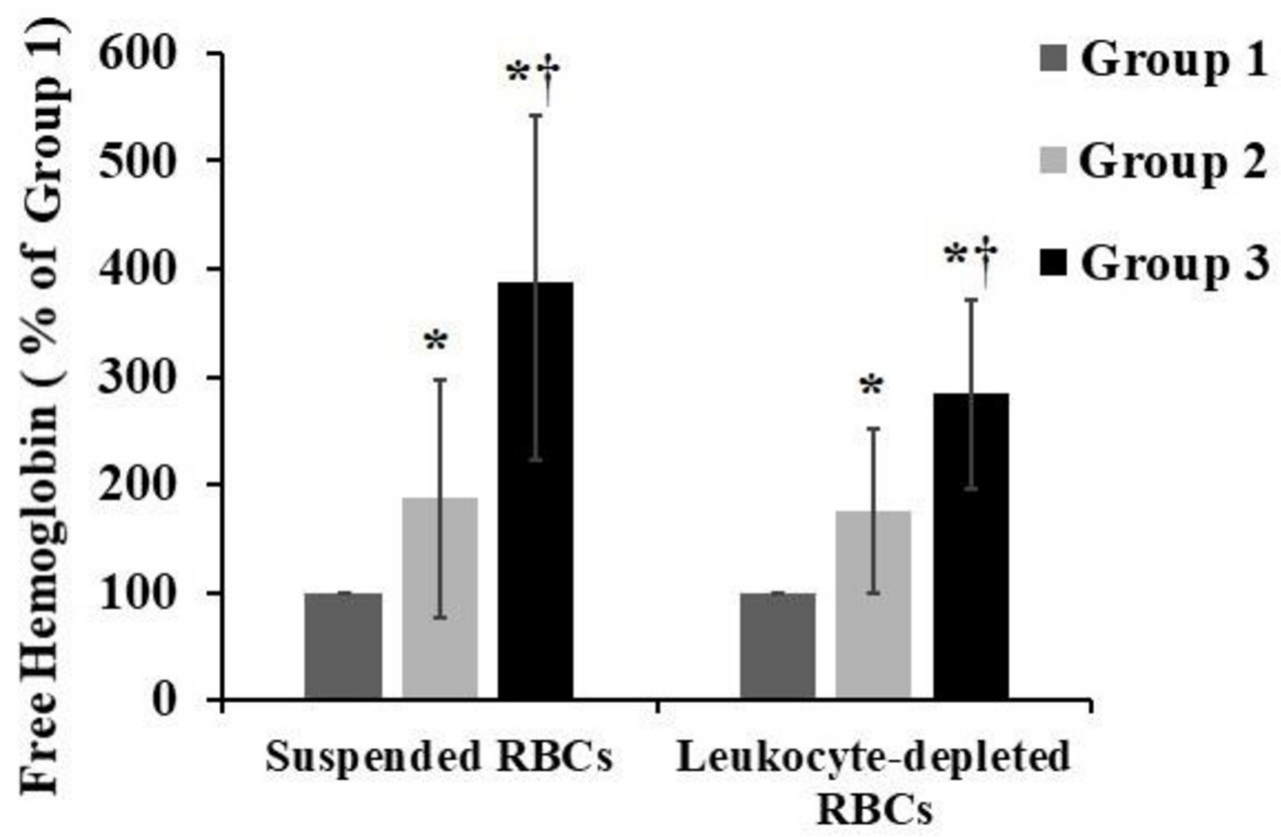
FHb contents were determined by a test kit according to the manufacturer’s instructions. Group 1 were the 1st day fresh-collected RBCs, group 2 was the 14th day RBCs with resting refrigerated storage, and group 3 was the 14th day RBCs after plateau transportation. The data represent mean ± SD from five RBCs samples in each group. *p < 0.05 compared to group 1; †p < 0.05 compared to group 2.

Among the suspended RBCs, the FHb concentration in group 1 was 87.35±43.98 mg/L, the concentration after storage for 14 days in Chengdu was 170.57±128.92 mg/L, and the concentration after plateau transportation was 321.79±186.00 mg/L. Among the leukocyte-depleted RBCs, the FHb concentration in group 1 was 144.81±116.45 mg/L, the concentration after storage for 14 days in Chengdu was 204.82±127.88 mg/L, and the concentration after plateau transportation was 331.16±195.37 mg/L. Different storage conditions, such as rutted roads and air transportation, alter aspects of RBC hemolysis. The FHb concentrations in suspended and leukocyte-depleted RBCs were increased, but after plateau transportation, the FHb concentration was significantly higher than that in Chengdu after storage for 14 days (p<0.05) (Fig 4).

### Analysis of the blood biochemical index

The pH values of the RBC suspensions in group 1, group 2 and group 3 were lower than 6.8, which was beyond the instrument detection range. The Na^+^ concentration of suspended RBCs stored in Chengdu for 14 days was 100.8±0.75 mmol/L, and after plateau transport, the Na^+^ concentration was 103.2±2.93 mmol/L, there was no significant difference between the two groups. The Na^+^ concentration of the leukocyte-depleted RBCs stored in Chengdu for 14 days was 100.33±0.47 mmol/L and that after plateau transport was <100 mmol/L (Table 1).

**Table 1:** The blood biochemical indexes over 14 days of storage.

Index	Suspended RBCs			Leukocyte-depleted RBCs		
	Group 1	Group 2	Group 3	Group 1	Group 2	Group 3
**pH**	<6.8	<6.8	<6.8	<6.8	<6.8	<6.8
**Na**^**+**^ **(mmol/L)**	106.2±2.04	100.8±0.75^*^	103.2±2.93	108±0.71	100.33±0.47^*^	<100
**K**^**+**^ **(mmol/L)**	5.96±1.42	>20	>20	5.55±0.59	>20	>20
**Glu (mmol/L)**	>27.8	21.84±1.09	22.1±1.66	>27.8	25.48±0.43	24.18±0.62
**Lac (mmol/L)**	5.8±0.54	>15	>15	4.6±0.37	>15	>15

Values shown are mean ± SD for n = 5 in each group.

*p < 0.05 compared to group 1

†p < 0.05 compared to group 2.

The K^+^ concentrations of the suspended RBCs and leukocyte-depleted RBCs at the beginning were 5.96±1.42 mmol/L and 5.55±0.59 mmol/L, respectively. After storage in Chengdu and plateau transport for 14 days, the K^+^ concentration was significantly increased, exceeding the instrument detection range at >20 mmol/L (p<0.05). The glucose consumption and lactic acid production in both RBC groups were significantly increased compared to that in group 1 (p<0.05) (Table 1).

### Analysis of 2, 3-DPG test results

**Fig 5:**
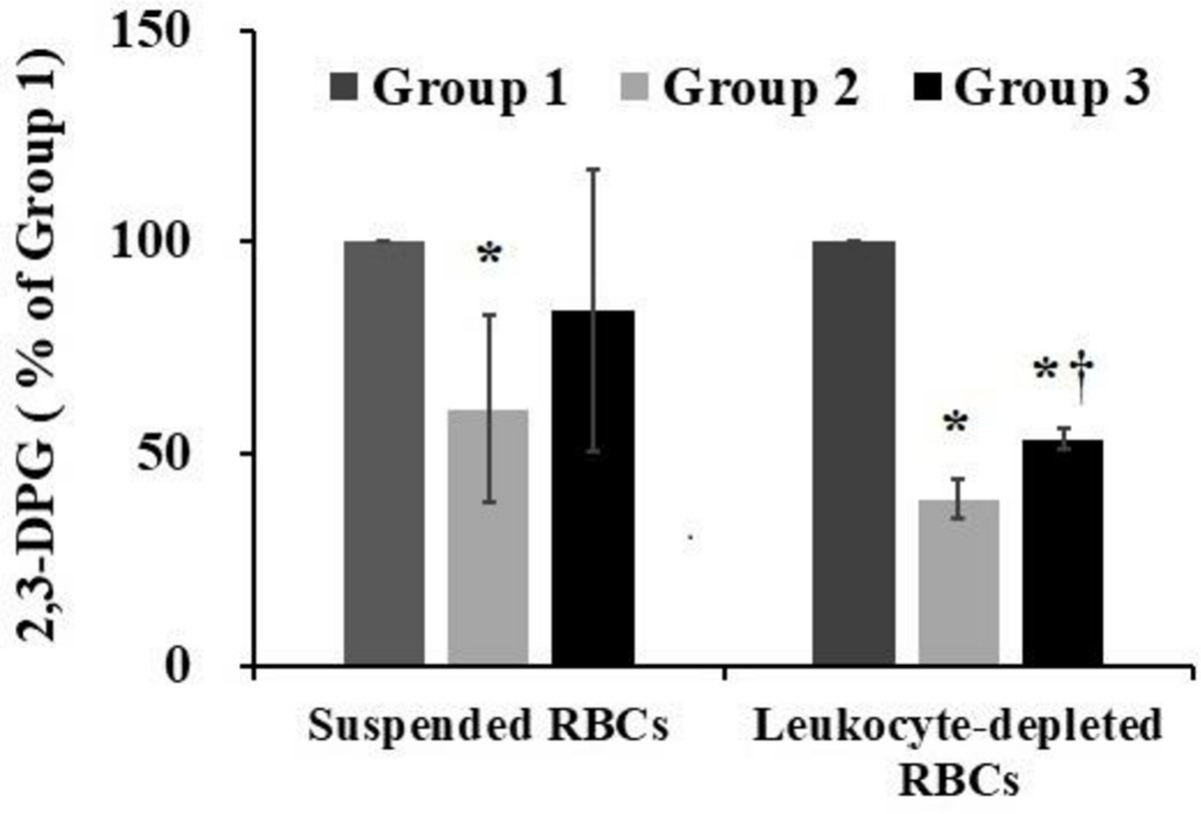
The 2,3-DPG contents were determined by a test kit according to the manufacturer’s instructions. Group 1 were the 1^st^ day fresh-collected RBCs, group 2 was the 14^th^ day RBCs with resting refrigerated storage, and group 3 was the 14^th^ day RBCs after plateau transportation. The data represent mean ± SD from five RBCs samples in each group. *p < 0.05 compared to group 1; †p < 0.05 compared to group 2.

The 2,3-DPG content in suspended RBCs was 245.57±35.44 nmol/L at the beginning of transportation. The 2,3-DPG content after storage for 10 days in Chengdu was 150.71±65.71 nmol/L and that after plateau transportation was 207.57.18±101.08 nmol/L, indicating a significant decrease. The 2,3-DPG content in leukocyte-depleted RBCs was 308.71±24.64 nmol/L before transportation, 121.57±15.93 nmol/L in group 2, and 165±15.43 nmol/L in group 3. The 2,3-DPG content in the two groups was decreased significantly compared to that in group 1 (p<0.05) (Fig 5).

### Hemorheologic index analysis

The hemorheologic indexes were affected by gender, age, diet, serum sodium, etc. Hematocrit (HCT) was the primary factor affecting the hemorheologic indexes[8]. Compared with group 2, whole blood viscosity, whole blood high and low cut relative index, RBC aggregation index, whole blood high and low cut reduction viscosity, RBC rigidity index, RBC deformation index and Carson viscosity were decreased in group 3. The plasma viscosity in group 2 was significantly decreased compared with that in group 3 (p<0.05) (Table 2).

**Table 2:** The hemorheologic indexes over 14 days of storage.

Index	Suspended RBCs			Leukocyte-depleted RBCs		
	Group1	Group2	Group3	Group1	Group2	Group3
**Shear Rate of Whole Blood Viscosity(1/S)**	8.51 ±1.00	9.40 ±0.55^*^	8.44±1.32^**†**^	8.35±0.37	9.57 ±0.47^*^	8.76± 0.25^**†**^
**Shear Rate of Whole Blood Viscosity(5/S)**	4.14 ±0.33	4.45 ±0.17^*^	4.05±0.42^**†**^	3.99 ±0.12	4.44 ±0.18^*^	4.12 ±0.13^**†**^
**Shear Rate of Whole Blood Viscosity(50/S)**	2.39 ±0.10	2.50±0.07^*^	2.31±0.11^*^^†^	2.26 ±0.05	2.43± 0.10^*^	2.30 ±0.09^**†**^
**Shear Rate of Whole Blood Viscosity(200/S)**	2.06 ±0.07	2.13 ±0.06	1.98±0.07^*^^†^	1.93±0.04	2.05±0.08^*^	1.95±0.08^**†**^
**Plasma Viscosity**	1.26 ±0.02	1.18 ±0.001^*^	1.24±0.01^*^^†^	1.28±0.03	1.20±0.02^*^	1.26±0.01^**†**^
**HCT**	0.22 ±0	0.22 ±0	0.22±0	0.22±0	0.22±0	0.22±0
**Whole Blood High Cut Relative Index**	1.64 ±0.09	1.80 ±0.05^*^	1.59±0.07^**†**^	1.50±0.04	1.71±0.08^*^	1.55±0.05^**†**^
**Whole Blood Low Cut Relative Index**	6.78 ±0.92	7.93±0.43^*^	6.80±1.13^**†**^	6.49±0.18	7.97±0.35^*^	6.96±0.20^*^^†^
**RBC Aggregation Index**	4.13 ±0.38	4.42±0.25^*^	4.26±0.52	4.31±0.17	4.66±0.20^*^	4.50±0.13^**†**^
**Whole Blood High Cut Reduction Viscosity**	32.98 ±4.64	37.33±2.47^*^	32.72±6.03^**†**^	32.05±1.51	38.07±2.10^*^	34.11±1.22^*^^†^
**Whole Blood Low Cut Reduction Viscosity**	3.64 ±0.43	4.28±0.27^*^	3.33±0.37^*^^†^	2.94±0.18	3.88±0.40^*^	3.14±0.32^**†**^
**RBC Rigidity Index**	2.89 ±0.40	3.62±0.23^*^	2.68±0.32^**†**^	2.29±0.16	3.23±0.36^*^	2.49±0.24^**†**^
**RBC Deformation Index**	0.81 ±0.08	0.95±0.04^*^	0.77±0.07^**†**^	0.68±0.04	0.88±0.07^*^	0.73±0.05^**†**^
**Carson Viscosity**	1.75 ±0.05	1.79±0.06	1.67±0.04^*^^†^	1.63±0.04	1.71± 0.07^*^	1.63±0.07^**†**^

Values shown are mean ± SD for n = 5 in each group.

*p < 0.05 compared to group 1

†p < 0.05 compared to group 2.

## Discussion

Due to the high altitude, low air pressure, rugged roads and wide coverage, higher requirements are placed on medical support in the plateau. As an important part of medical support, blood availability is particularly important in the development of plateau areas[9]. At present, high-altitude areas, such as Lhasa in Tibet, have not carried out component blood transfusions, and there is no experience and data for preparing and preserving suspended RBCs. Therefore, the promotion of storage and transportation of RBC products in plateau areas is a problem to be solved[1]. National and international blood bank authorities have noted that the US Army research and development efforts in providing new blood products and improving blood safety operate on the cutting edge of technology and are transformational for the global blood industry. Military conflicts have many unwanted consequences; however, in times of conflict, one positive aspect is the identification of novel solutions to improve the safety and efficacy of the blood supply[10]. The five major elements (collection, processing, storage, transportation, and transfusion activities) must come together under the direction of the Army, Navy, and Air Force blood programs to maintain the blood flow from arm to arm and donor to recipient. In other words, blood storage and transportation play a very important role in blood products.

In every military conflict, there have been numerous innovations that have improved not only military health care but also the availability of commercial blood products (whole blood, freeze-dried plasma (FDP), and frozen RBCs) and devices (plastic blood bags, low-cost shipping containers, and ASs (additive solutions)) [11]. In austere environments, the military does not have the luxury of building state-of-the-art facilities with the entire infrastructure of modern hospitals. As a result, all military requirements include an examination of the logistic support needed for a new capability. Military blood banks must operate in any environment worldwide and adapt to new challenges by searching for new capabilities to meet current and future requirements.

Military donor centers have a requirement to ship blood products that support the deployed warfighters within 4 days of collection[10]. The results in this study reveal the effects of transporting blood in a phase-change material transport container. The storage times of group 2 and group 3 were extended to 14 days, and both RBC counts and HGB were decreased compared with those in group 1. The FHb content showed an increasing trend, but the hemolysis rate was <0.8%. These effects still meet the national standard (Table 3, GB 18469–2012 whole blood and component blood quality requirements)[12]. The phase-change material transport container can maintain the blood in the container for a long time (1–10°C) under complex high altitude conditions, such as transportation by road, continuous ambient temperature and altitude change, and car oscillation frequency.

**Table 3:** Quality control and requirement of suspended RBCs and leukocyte-depleted suspended RBCs (GB18469-2012).

Quality Control	Requirement	
	Suspended RBCs	leukocyte-depleted suspended RBCs
**Appearance**	Normal color, no hemolysis, clots, bubbles, intact bags, thermal catheter≥35cm	
**Capacity**	Labelled amount(ml)±10%	
**Hemolysis rate at the end of storage**	<0.8%	
**Sterile experiment**	—	
**Hematocrit(HCT)**	0.45~0.60	0.45~0.60
**Hemoglobin content**	≥20g/200ml; ≥30g/300ml; ≥40g/400ml.	≥18g/200ml; ≥27g/300ml; ≥36g/400ml.
**Residual amount of WBC**		≤2.5×10^6^/200ml; ≤3.8×10^6^/300ml; ≤5.0×10^6^/400ml.

The K^+^ contents of RBCs after transportation were increased rapidly, and the Na^+^ contents decreased slightly. RBCs may have leakage of K^+^ during transportation from Lhasa to Chengdu. Although K^+^ is not listed as an assessment index in GB18469-2012, the influence of high K^+^ factors on the recipient should be considered in special environments. The content of glucose was at a high level at the beginning and beyond the instrument detection range. The glucose consumption and lactic acid were increased, indicating that the RBCs underwent active anaerobic respiratory metabolism during storage. Notably, 2, 3- DPG is an intermediate in RBC metabolism and is considered an important indicator of the oxygen-carrying ability of RBCs[13,14]. The 2,3-DPG contents after transportation were decreased, indicating that the oxygen-carrying capacity of RBCs was decreased. The results showed that storage damage of RBCs gradually occurred with prolonged storage time[15].

Studies have shown that hemorheologic indexes were decreased under the same hematocrit in a plateau environment. Reducing RBC aggregation is also an effective way to regulate hemorheologic indexes[16]. In the regulation of hemorheologic indexes in people in the plateau, the weakening of RBC aggregation can also lead to a decrease in hemorheologic index levels. The main reasons are as follows: (1) The plasma fibrinogen content is reduced under high altitude hypoxic conditions, while fibrinogen is increased in RBC aggregation and plays an important role as a “bridge”. Less fibrinogen weakens RBC aggregation. (2) Long-term hypoxia caused changes in the blood coagulation mechanism, showing a decrease in the number of coagulation factors and platelets, the hyperfibrinolytic system, and the platelet adhesion rate, and these changes are conducive to reducing RBC aggregation. (3) Hypoxia affected the energy metabolism and charge distribution of erythrocyte membranes and affected the aggregation function of RBCs. In addition, the morphological changes of RBCs also play an important role in the regulation of whole blood viscosity. In a long-term hypoxic environment, there will be some changes in the morphology of RBCs, mainly due to the increased volume. Studies have shown that the greater the ratio of erythrocyte membrane surface area / hematocrit, the stronger the deformability of RBCs.

The RBC aggregation index is an indicator that roughly reflects the degree of RBC aggregation. The larger the value, the more easily the RBCs aggregate[16]. The increase in the RBC aggregation index is the main reason for the increase in low-cut whole hemorheologic indexes. The RBC rigidity is a parameter that roughly reflects RBC deformability, including the RBC rigidity index and RBC deformation index. The larger the value, the worse the RBC deformability. Under the same hematocrit, the hemorheologic indexes of group 2 and group 3 were increased, but the hemorheologic indexes of group 3 were less than those of group 2. This result might be caused by excessive RBC damage in group 3 after storage, thereby reducing the hemorheologic indexes.

The effects of transport on the RBCs in the plateau environment certainly existed. The FHb levels in group 3 were significantly higher than those in group 2. In the process of blood transportation, the phase-change material transport container is small in size, light in weight, and has no power supply requirement. The heat preservation time was longer than that of the ordinary passive refrigerator, overcoming the short time of the ordinary foam insulation container[17,18]. This container is of great value in the implementation of blood transfusion and treatment during natural disasters or in wartime.

## Supporting information

S1 Data (XLSX)
